# Haematopoietic Stem Cell Transplantation for the Treatment of Multiple Sclerosis: Recent Advances

**DOI:** 10.1007/s11910-023-01290-2

**Published:** 2023-08-17

**Authors:** Alice Mariottini, Eleonora De Matteis, Maria Teresa Cencioni, Paolo A. Muraro

**Affiliations:** 1 Department of Brain Sciences, Imperial College London, London, UK; 2 Department of Neurosciences, Drug and Child Health, University of Florence, Florence, Italy; 3 Department of Biotechnological and Applied Clinical Sciences, University of L’Aquila, L’Aquila, Italy

**Keywords:** Haematopoietic stem cell transplantation, Multiple sclerosis, Alemtuzumab, Reconstitution, Biomarker

## Abstract

**Purpose of Review:**

Autologous haematopoietic stem cell transplantation (AHSCT) is increasingly considered a treatment option for patients with multiple sclerosis (MS), an autoimmune demyelinating and degenerative disease of the central nervous system (CNS). AHSCT persistently suppresses inflammation and improves the disease course in large proportions of patients with relapsing–remitting (RR) MS. Aim of this article is to review the relevant new knowledge published during the last 3 years.

**Recent Findings:**

Laboratory studies reported confirmatory and new insights into the immunological and biomarker effects of AHSCT. Retrospective clinical studies confirmed excellent outcomes in RRMS, showing possible superior effectiveness over standard therapies and suggesting a possible benefit in early secondary progressive (SP) MS with inflammatory features. New data on risks of infertility and secondary autoimmunity were also reported.

**Summary:**

Further evidence on the high effectiveness and acceptable safety of AHSCT strengthens its position as a clinical option for aggressive RRMS. Further research is needed to better define its role in treatment-naïve and progressive forms of MS, ideally within randomised clinical trials (RCTs).

## Introduction 

Over the last 10 years, autologous haematopoietic stem cell transplantation (AHSCT) has been increasingly adopted for the treatment of people with aggressive forms of multiple sclerosis (MS) [1], an autoimmune demyelinating and degenerative disease of the central nervous system (CNS) that may lead to irreversible disability [2]. AHSCT involves the ablation of the immune system and its reconstitution, which appear to restore immune tolerance [3] and induce long-term suppression of new focal inflammation (relapses and new/enhancing lesions at magnetic resonance imaging (MRI)) in most of the treated individuals. Stabilisation, or even improvement of the course of MS, is often achieved when AHSCT is performed in early relapsing–remitting (RR) MS, whereas outcomes are uncertain in progressive forms of MS, i.e. primary progressive (PP) and secondary progressive (SP) MS, and are poor in advanced stages of any form. Based on the results of several uncontrolled studies and one randomised clinical trial (RCT) proving its superior effectiveness compared to selected approved disease-modifying therapies (DMTs), AHSCT was endorsed as a ‘clinical option’ for the treatment of RRMS refractory to conventional DMTs by the European and the American Societies for Blood and Marrow Transplantation [4, 5]. The use of intermediate-intensity protocols, namely either the lymphoablative cyclophosphamide (Cy) plus anti-thymocyte globulin (ATG) or the myeloablative BEAM (carmustine, cytarabine, etoposide and melphalan) plus ATG, is currently recommended in MS [4].

Several studies of AHSCT in MS have been recently published, adding valuable information to the current knowledge on its mechanisms of action, effectiveness and safety. Aim of the present review is to highlight and discuss the main novelties introduced in this field over the last 3 years.

## Materials and Methods

The PubMed database was searched using the MeSH terms ‘hematopoietic stem cell transplantation’ and ‘multiple sclerosis’, filtering for studies on humans published in the period January 1st, 2020–May 15th, 2023. After the exclusion of reviews, meta-analyses and manuscripts written in non-English language, 37 studies were included (Tables 1 and 2).

**Table 1:** Main baseline, disease characteristics, effectiveness, and safety outcomes of the most recent real-world studies on AHSCT

Study, year (Ref. No.)	Treatment	*N* of patients	Type of conditioning	Follow-up duration (months ± IQR/SD or range)	Age (years ± IQR/SD or range)	MS type, *N* (%)	Baseline EDSS (median ± IQR)	Disease duration (years ± IQR/SD or range)	NEDA rate and time assessed	TRM (%)
Das et al., 2021 [32]	AHSCT	20	4 (20) Bu-Cy-ATG; 4 (20) BEAM-ATG; 12 (60) Cy-ATG	30 (12–118)	28 (17–47)	20 (100) aggressive MS	5 (1.5–9.5)	5 (1–20)	100% at mean FU	0
Nicholas et al., 2021 [24]	AHSCT	120	2 (2) BEAM-ATG; 118 (98) Cy-ATG	21 (6–85)	42.3 (± 8.8)	58 (48) RRMS; 40 (33) SPMS; 22 (18) PPMS	6.0 (5.5–6.5)	8.9 (± 5.3)	65% at 2 y	2.5
Boffa et al., 2021 [25]	AHSCT	210	74.8% BEAM-ATG; 4.8% BEAM; 8.1% Cy-ATG; 1% others	74 ± 60	34.8 (± 8.6)	122 (58) RRMS; 88 (42) PMS	6.0 (4.5–6.5)	11.0 (± 6.7)	57.9% at 5 y	1.4
Burt et al., 2022 [22••]	AHSCT	507	376 (74) Cy-ATG; 63 (12) Cy-ATG-RTX; 26 (5) Cy-ALZ; 46 (9) Cy-ATG-IVIg	31	37 (± 8.01)	414 (81) RRMS; 93 (19) SPMS	4.1 (± 1.48)	7.2 (± 5.4)	NA	0.19
Kvistad et al., 2022 [23]	AHSCT	104	104 (100) Cy-ATG	39 (1–95)	30 (10–58)	104 (100) RRMS	3 (0–6.5)	5.8	81% at last FU	0
Zhukovsky et al., 2020 [34•]	AHSCT	69	69 (100) Cy-ATG	36	30 (26–37)*	69 (100) RRMS	3 (2–4)*	6.4 (± 5.7)	88% at 3 y	0
	ALZ	75		36	35 (30–41)	75 (100) RRMS	2 (1–2.5)	7.0 (± 5.4)	37% at 3 y	0
Boffa et al., 2020 [35]	AHSCT	25	25 (100) BEAM-ATG	50.9 (± 48.2)*	32.1 (9.9)	25 (100) RRMS	6 (4.5–7)*	9.5 (± 5.4)	75% at last FU	0
	ALZ	32	–	29.3 (± 11.3)	35.1 (8)	32 (100) RRMS	3 (1–4)	7.2 (± 5.9)	56% at last FU	0
Häußler et al., 2021 [36•]	AHSCT	19	19 (100) BEAM-ATG	57 (29–149)*	35.1 (10.0)	12 (63.1) RRMS; 3 (15.8) PPMS; 4 (21.1) SPMS	4.5 (± 2.0)	5.4 (± 4.2)*	62% at last FU	0
	ALZ	21	–	27 (11–52)*	39.0 (9.8)	16 (76.2) RRMS; 5 (23.8) SPMS	4.7 (± 1.7)	11.3 (± 6.8)	40.2% at last FU	0
Mariottini et al., 2021 [31]	AHSCT	31	31 (100) BEAM-ATG	99 (24–238)	39.3 (± 7.27)*	31 (100) SPMS	5.9 (± 0.87)	13.7 (± 5.28)	45%§ at 5 y	0
	Cy	62	–	91 (7–285)	42.8 (± 7.09)*	62 (100) SPMS	5.7 (± 1.01)	13.8 (± 6.73)	36%§ at 5 y	0
Boffa et al., 2023 [30]	AHSCT	79	64 (81) BEAM-ATG; 11 (14) Cy-ATG; 3 (4) Thiothepa-ATG; 1 (1) other	5.6 (2.2–11.1)	39 (± 7.8)	79 (100) SPMS	6.2 (± 0.9)	13.7 (± 6.8)	NA	1.3
	DMTs†	1975	–	3.9 (1.7–6.4)	39 (± 7.8)	1975 (100) SPMS	6.2 (± 0.9)	13.7 (± 6.6)	NA	NA
Kalincik et al., 2023 [37]	AHSCT^a^	167	43 (26%) HI; 49 (29%) II-myeloablative; 64 (38%) II-lymphoablative; 11 (7%) L/II	4.07 (± 2.61)	35.0 (± 8.8)	167 (100) RRMS	4.01 (± 1.73)	7.88 (± 5.43)	NA	1 (0.6%)
	Fingolimod^a^ matched	2558	–	2.80 (± 2.24)	38.4 (± 10.0)	2558 (100) RRMS	2.35 (± 1.61)	9.56 (± 7.17)	NA	NA
		769		2.84 (± 2.43)	35.3 (± 9.4)	769 (100) RRMS	3.75 (± 1.82)	8.17 (± 6.07)	NA	NA
	Natalizumab^a^ matched	1490	–	2.50 (± 2.14)	36.8 (± 9.8)	1490 (100) RRMS	2.91 (± 1.75)	8.74 (± 6.92)	NA	NA
		730		2.51 (± 2.22)	36.0 (± 9.0)	730 (100) RRMS	3.88 (± 1.92)	; 8.17 (± 6.22)	NA	NA
	Ocrelizumab^a^ matched	700	–	1.64 (± 0.98)	41.8 (± 11.2)	700 (100) RRMS	3.03 (± 1.89)	10.89 (± 7.79)	NA	NA
		343		1.52 (± 0.94)	37.1 (± 10.6)	343 (100) RRMS	3.58 (± 1.87)	8.48 (± 7.34)	NA	NA

*AHSCT* autologous haematopoietic stem cell transplantation; *ALZ* alemtuzumab; *ARR* annual relapse rate; *ATG* anti-thymocyte globulin; *BEAM* bis-chloroethylnitrosourea (BCNU), etoposide, cytosine arabinoside (ARA-C), and Melphalan; *Cy* cyclophosphamide; *DMTs* disease-modifying treatments; *EDSS* expanded disability status scale; *HI* high intensity; *II* intermediate intensity; *IQR* interquartile range; *LI* low intensity; *MS* multiple sclerosis; *NA* not available; *N* number; *RRMS* relapsing–remitting multiple sclerosis; *SD* standard deviation; *SPMS* secondary progressive multiple sclerosis; *TRM* transplant-related mortality

^*^Statistical significance; §NEDA-2; †beta interferons, azathioprine, glatiramer acetate, mitoxantrone, fingolimod, natalizumab, methotrexate, teriflunomide, cyclophosphamide, dimethyl fumarate and alemtuzumab

^a^Population before propensity score matching (donor pool)

**Table 2:** Main characteristics and findings of the studies evaluating biomarkers in patients after AHSCT

Study, year	*N* of cases	Type of conditioning, *N* (%)	FU duration	Type of biomarker	Site	Main findings
Larsson et al., 2019 [13••]	46 patients	15 (32) BEAM-ATG; 31 (68) Cy-ATG	2 years	Cell count, IgM and IgG index, BOCs, NfL	CSF	Mononuclear cells, CSF/serum albumin ratio, IgM and IgG index, NfL, and BOCs ↓
Thebault et al., 2020 [14]	22 patients; 28 controls	22 (100) Bu-Cy	1 year	NfL, GFAP	Serum	At 3 and 6 months, NfL and GFAP ↑ and correlated with grey matter atrophy, EDSS worsening, and Bu dose; at 12 months, NfL ↓ below baseline levels, GFAP ↓ at levels similar to baseline
Zjukovskaja et al., 2022 [17]	43 patients; 31 controls	43 (100) Cy-ATG	3.9 years (IQR 2.2–4.3)	NfL, MBP, GFAP	CSF	NfL and MBP ↓ and patients with NEDA had lower NfL and MBP levels at baseline; GFAP remained stable and baseline levels did not differ between those with or without NEDA
Ruder et al., 2022 [15]	11 patients, CSF; 32 patients, serum	32 (100) BEAM-ATG	NA	NfL, GFAP	CSF, serum	In CSF at 24 months, NfL and GFAP ↑; in serum, NfL and GFAP ↑ at 1 month and ↓ to normal levels up to the end of FU
Mariottini et al., 2022 [18]	38 MS-AHSCT; 22 SPMS (controls); 19 healthy controls	36 (95) BEAM-ATG; 2 (5) melphalan-carmustine ATG or BEAM without melphalan	49 months (range 24–153)	NfL	Serum	At month 6 NfL were similar to baseline; at month 24, NfL ↓ and were similar to SPMS (controls) but still higher than healthy controls

*AHSCT* autologous haematopoietic stem cell transplantation; *ATG* anti-thymocyte globulin; *BOC* oligoclonal bands; *BEAM* bis-chloroethylnitrosourea (BCNU), etoposide, cytosine arabinoside (ARA-C), and melphalan; *Cy* cyclophosphamide; *GFAP* glial acidic fibrillary protein; *IQR* interquartile range; *MBP* myelin basic protein; *MS* multiple sclerosis; *NfL* neurofilament; *NA* not available; *N* number; *SPMS* secondary progressive multiple sclerosis

## Mechanism of Action and Biomarkers

### Immune Reconstitution 

The pathogenesis of MS has been attributed to autoreactive T cells that after becoming activated in peripheral lymphoid organs (e.g. lymph nodes) migrate to the CNS where they cause inflammation [6]. In this section, we update previous review articles covering immune reconstitution in patients with MS after HSCT [3, 7].

T cell immune reconstitution was recently investigated up to month 24 after treatment in 27 MS patients receiving AHSCT with the BEAM-ATG regimen and anti-CD20 treatment before AHSCT (78%), including as controls healthy people (HC) and 2 untreated RRMS and PPMS, matched for age and sex [8]. CD4 + T cells recovered slowly compared to CD8 + T cells, with a reduction in the CD4/CD8 ratio for almost 24 months. An increase of CD127^low^ CD25 + Foxp3 + regulatory T cells (Treg) expressing CD39 was observed from 1 to 3 months after AHSCT, although the absolute number of Treg was always decreased after AHSCT. CD4 + T cells increased the expression of programmed cell death protein 1 (PD1) and major histocompatibility complex II cell surface receptor (HLA)-DR, a marker associated to T cell activation, up to 24 months after AHSCT. In the CD4 + T cell compartment, the frequency of naïve and central memory (CM) cells decreased, whereas effector memory (EM) increased up to 24 months after AHSCT compared to baseline. Within naïve CD4 + T cells, recent thymic emigrant (RTE) decreased significantly at 3 months but increased at 12 months after AHSCT. Double positive CD4 + /CD8 + T cells reduced until 6 months supporting the notion that thymus reactivation requires months to years. Early after AHSCT, senescent and exhausted antigen-experienced EM expanded, whereas naïve, CM and terminally differentiated effector memory cells re-expressing CD45RA (TEMRA) reconstituted slowly. Similar processes were observed in CD8 + T cells. Adopting T cell receptor beta (TCRβ) chain sequencing and TCR clonotyping, expanded EM cells in early AHSCT were found to be derived from memory T cells surviving to the conditioning regimen. Those EM cells proliferated less than new EM CD4 + T cells and showed a non-proinflammatory phenotype. In line with previous reports, reactivity towards myelin oligodendrocyte glycoprotein (MOG), myelin basic protein (MBP) and proteolipid protein (PLP) was stable late after AHSCT, whereas it was increased against Epstein-Barr nuclear antigen (EBNA) 1, mostly in patients with Epstein-Barr virus (EBV) reactivation.

Natural killer (NK) and B cells recovered faster than CD3 + T cells. Three flow cytometry panels were used to investigate NK and innate-like T cell immune reconstitution dynamics [9]. A significant increase of CD56^bright^ NK cells with immunoregulatory functions was observed at 1 month post AHSCT up to 2 years, whereas CD56^dim^ with cytotoxic function increased at 1 month then declined with slow recovery over 2 years. Mucosal-associated invariant T (MAIT) cells, γδT cells and NK(-like) T cells decreased after AHSCT and remained depleted for at least 1 year. The expression of several tissue-homing receptors was investigated on innate-like T cells to examine the capacity of those cells to enter specific tissues. Lower percentages of innate-like T cells expressed CD161 at pre- and post-AHSCT. CCR6 was reduced on CD8 + T cells after AHSCT. CD8 + CCR6 + T cells decreased for at least 2 years post AHSCT, while CD4 + CCR6 + T cells reduced transiently to recovering later. After AHSCT, a slight decrease in the percentage of CD56^bright^ NK cells CD161 was observed at 1 year, whereas CD56^dim^ NK cells showed a significant decrease in CD161 expression at 1 month. The absolute number of Vd2 γδ T cells decreased after AHSCT.

In another recent study, longitudinal multidimensional cytometry and immunoglobulin heavy chain (IgH) repertoire sequencing were used to investigate the B cell immune reconstitution in 20 MS patients receiving AHSCT with BEAM-ATG regimen and anti-CD20 treatment before and 1, 3, 6 and 12 months after AHSCT and compared to HC [10•]. B-lymphocyte number recovered at 3 months and remained increased at 1 year post AHSCT. Transitional immature B cells represented the largest B cell population at 1 month after AHSCT, the percentage decreasing over the following months. Mature naïve B cells rose after 3 months and remained elevated over the study period. Within the memory compartment, switched memory B cells were higher compared to HC at pre- and at 1 month post AHSCT reducing HC levels at 3 months post AHSCT. The proportion of switched plasma cells did not change over the study period. The IGHJ genes closer to the recombination site (IGHJ1, 2 and 3) were overexpressed in the repertoire of B cells reconstituted early post-transplant, whereas B cells using IGHJ5 and IGHJ6 (located farther from the recombination site) reconstituted later. IGHV genes closer to recombination sites were overexpressed on early naïve repertoire but not in early reconstituted antigen-experienced repertoire. Mutation analyses on the Ig repertoire showed an elevated number of mutations on all switched isotypes until 3 months post AHSCT followed by a decline and increase at 12 months, supporting the persistence of antigen-experienced memory populations. Shannon diversity index showed a significant reduction in diversity in all memory repertoires and at all time points after AHSCT. Repopulation of naïve and unswitched memory B subsets was significantly delayed in patients showing early cytomegalovirus (CMV) reactivation and remained below the level of patients without CMV reactivation throughout follow-up. In those patients, sequence cluster overlap was observed between pre- and post-AHSCT suggesting the expansion of persistent memory B cell clones. It is not clear whether these clusters contain CMV-specific clones.

**Fig. 1:**
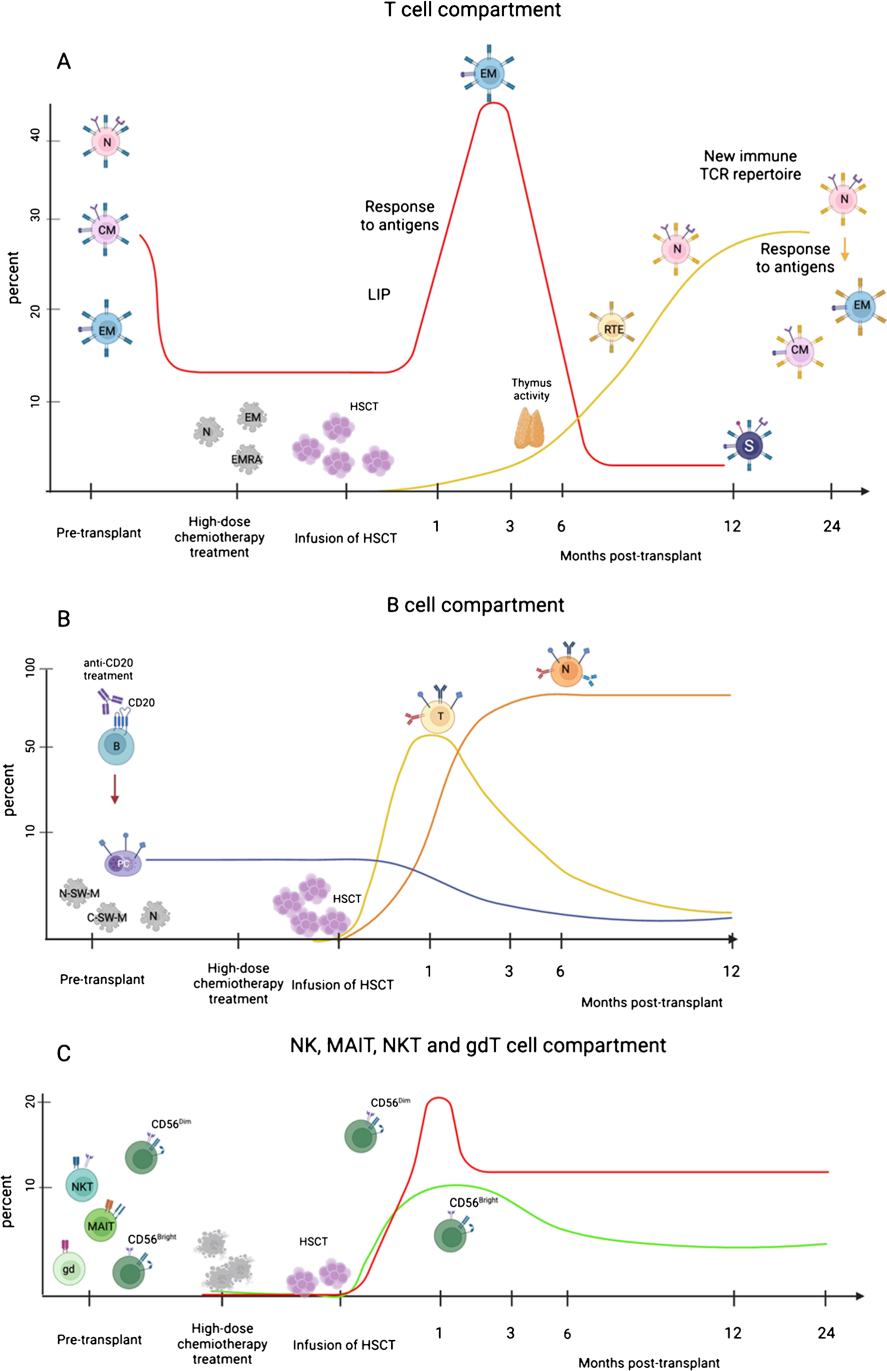
Immune reconstitution after HSCT. **A** T cell dynamics. T cell numbers reconstituted slowly and remained under the baseline during follow-up. Different stages of T cell differentiation were defined by CCR7 and CD45RA expression. Naive (N) T cells recovered slowly by thymus-dependent reconstitution only after 6 months post HSCT. Effector memory (EM) T cells increased rapidly after HSCT stimulated by lymphopenia-induced proliferation (LIP) and antigen responses and remained above the baseline during the period study. Most of the EM differentiated from central memory (CM) T cells resistant to high-dose chemotherapy treatment. The persistent EM proliferated less than newly emerging T cells and differentiated in terminally differentiated memory cells (EMRA) with senescent and exhausted phenotype (S) (CD57 + , CD27 − , CD28 − , PD1 +) with reduced proinflammatory potential. Naïve T cells eventually differentiated in EM and CM with new TCR repertoire. **B** B cell dynamics. B cells were depleted by anti-CD20 treatment before HSCT. The subsets of B cells were defined by the expression of CD24, CD24 and CD38. Substantial proportion of early B cells post-HSCT was plasma cells (PCs). Transitional B cells (T) increased at 1 months after HSCT and decreased in the following months, while mature B cells rose and remained higher during the period study. Memory B cells generated slowly and remained below the baseline at 1-year follow-up. **C** Dynamics of NK and innate-like T cells including $\gamma \delta$ T cells, mucosal-associated invariant T (MAIT) and NK(-like) T cells. NK and innate-like T cell subsets were identified by the expression of CD56, CD3 $\gamma \delta$ /$\alpha \beta$/V*a*7.2 TCR, CD161 and IL18R. NK cells include 2 subsets CD56^bright^ with immunoregulatory functions and CD56^dim^ with high cytotoxic functions. CD56^bright^ cells increased after HSCT, while CD56^dim^ increased after 1 month and declined to recovery slowly during the 2-year follow-up. MAIT, $\gamma \delta$ T and NK(-like) T cells decreased after HSCT and remained below the baseline

Overall, these studies expand the pre-existing knowledge on adaptive and some innate cell population reconstitution after AHSCT with BEAM-ATG and Cy-ATG conditioning, suggesting that some mechanisms of immune reconstitution after AHSCT are shared by these conditioning protocols. Consistent with previous reports, they demonstrate a substantial degree of renewal of adaptive immunity, consistent with the notion of ‘immune resetting’, although evidence of association of the observed immunological changes with clinical efficacy is limited, partly due to the overall high efficacy of the treatment strategy. A summary of the dynamics of ablation and reconstitution of relevant lymphocyte subpopulations during and up to 24 months after AHSCT, as reported in the two studies reviewed here, is presented in Fig. 1.

### Biomarkers 

Few and heterogenous studies examined neuroimaging and soluble biomarkers of neuroinflammation and neurodegeneration in blood and cerebrospinal fluid (CSF) after the treatment (Table 2).

#### Neurofilaments and Glial Fibrillary Acidic Protein

Recent reports showed that CSF neurofilament light chains (NfLs) (validated biomarker for MS activity) [11, 12] gradually decrease after AHSCT [13••, 14] despite an initial non-significant increase [15]. Conversely, CSF glial fibrillary acidic protein (GFAP; released during astrocyte activation and astrogliosis following inflammation and neurodegeneration) [16] remained high after AHSCT [17]. At baseline, 67–72% of patients had pathological values of NfL [13••, 17]; the median value of NfL steadily decreased over time, and almost all patients had normal values at 4 and 5 years after AHSCT [13••, 17].

Serum NfL (sNfL) and GFAP increased between 1 and 6 months after AHSCT likely due to chemotherapy-related neurotoxicity and gradually decreased to levels lower or comparable to pre-treatment in the long term [14, 15, 18]. SNfL increase was observed in patients treated with both high- and moderate-intensity conditioning regimens [14, 15, 18], and a correlation between sNfL and the total busulfan dose was suggested [14]. Moreover, sNfL increase correlated with transient worsening of post-treatment Expanded Disability Status Scale (EDSS) score and MRI brain volume loss (BVL) and was associated with cognitive deterioration, which might explain the transient ‘chemo fog’ often reported after AHSCT [14]. Disease forms did not affect sNfL decrease in the long term, and sNfL levels either at baseline or follow-up did not correlate with disability accrual or normalisation of the MRI rate of BVL [18].

#### Myelin Basic Protein, Oligoclonal Bands and IgG and IgM Index

Other CSF biomarkers of neuroinflammation were reduced after AHSCT such as MBP—a marker of demyelination—that was abnormal in 63% of patients at baseline [17]. Protein levels decreased up to normal levels in 88% of patients at 5 years post AHSCT [17].

Similarly, a gradual decrease in IgG production occurred over time, and patients with positive IgG oligoclonal bands (OCB) decreased from 98 to 74% at the last follow-up [13••]. Proportion of patients with normal IgG index significantly increased from 30 to 54% after AHSCT and those with normal IgM index from 21 to 58% [13••].

#### Cytokines and Chemokines

AHSCT is expected to shift the MS cytokines and chemokine inflammatory profile towards a non-inflammatory profile [19, 20]. Wiberg et al. showed that several serum proteins expressed by blood cells decreased shortly after the conditioning in patients treated with Cy-ATG; protein levels came back to normal at 3 months from AHSCT [19]. Other serum proteins expressed in various tissues (i.e. CXCL9, CX3CL1, MCP-1 and CXCL10 with chemotactic effects for T cells and monocytes or proteins involved in cell differentiation and growth) dropped after mobilisation, increased during the conditioning and gradually reached normal levels within 3 months from HSC reinfusion [19]. Ruder et al. showed that CSF levels of CXCL9, 10 and 13 did not significantly change after AHSCT despite CXCL9 and 10 increased 24 months after the treatment possibly due to early viral reactivation or other treatment-related infections [15]. Serum CXCL10 significantly increased 1 month after AHSCT and normalised at 3 months in the overall cohort and, more remarkably, in those with CMV reactivation compared with those with EBV reactivation. In addition, CXCL10 increase was associated to changes in T helper phenotypes of CD4 + EM T cells—there was an increase in Th1 and a decrease in Th2 [15]. Another study including patients treated with a low-intensity regimen showed a significant decrease of serum IL-21 and IL-22 released by NK, Th1 and Th17 and an increase of CCL2 and CCL4 with an unclear role in MS 14 days after AHSCT compared with 14 days prior to the treatment [20].

#### Brain Atrophy

Recent studies showed an increased rate of BVL, ranging between − 1.15 and − 2.18 within 12 months after HSCT in patients treated with BEAM-ATG or Bu-Cy [14, 18, 21]. BVL rate subsequently normalised and since month 24 after AHSCT became like normal ageing values. This initial ‘pseudoatrophy’ might be due to the neurotoxicity of chemotherapies or the resolution of neuroinflammation. Twelve-month BVL of grey and white matter was higher in patients with enhancing lesions before AHSCT, while white matter BVL was also higher in those with a higher number of T1-weighted lesions before AHSCT (i.e. amount of tissue irreversibly injured before treatment). White matter BVL, thus, seems to be related to pre-existing tissue damage [21]. In the long term, BVL rates were similar among subgroups [21] and among those with RRMS and SPMS [18].

## Efficacy

### Cohort Studies 

Four studies reported AHSCT outcomes in large real-life cohorts, each including > 100 MS patients, 507 as the largest [22••]; the protocols predominantly used were Cy-ATG [22••, 23, 24] or BEAM-ATG [25] (Table 1). One study focused on early RRMS (including 11.5% treatment-naïve) [23], one on RRMS and early SPMS [22••], and the remaining two included all the MS phenotypes, with a minor proportion of PPMS cases [24, 25].

All these studies confirmed the high effectiveness of AHSCT in RRMS: relapse-free survival (RFS) was approximately 80% at year 5 across all cohorts, whereas progression-free survival (PFS) ranged from 63% [24] to 95% at year 4 [22••] and 85.5% at year 5 [25]. Such variability in PFS could be attributable, at least in part, to (i) different baseline characteristics and (ii) the possible inclusion of variable proportions of cases with early SPMS, due to the known uncertainty and delay in diagnosing the transition from RRMS to SPMS in real life [26]. A significant improvement in median EDSS score was reported after AHSCT [24, 25]; it was substantial (mean 1.7 EDSS point) and sustained up to year 5 in one study, where it was observed to persist at least 2 years from transplant and irrespective of baseline score and type of lymphoablative regimen used [22••]. No evidence of disease activity (NEDA)-3 survival ranged from 48% at year 4 [24] to 62% at year 5 [25] and 81% at a median follow-up of 39.5 months [23]. The effectiveness of AHSCT in RRMS was sustained for up to 10 years, with RFS, PFS and NEDA-3 of 63.5%, 71% and 40.5%, respectively [25]. Interestingly, the use of BEAM-ATG compared to other conditioning regimens (lymphoablative in most cases) was independently associated with a lower risk of relapses and MRI inflammatory activity in both RRMS and progressive MS. On the other hand, protocol intensity seemed not to influence PFS in either form [25], suggesting that pathogenetic mechanisms underlying disease progression may not be differentially affected.

PFS in progressive MS was generally lower than in RRMS, ranging from 66% at year 4 [22••] to 71% at year 5 [25], except for one study, where PFS was similar, but EDSS change after transplant differed between the two groups, in favour of RRMS [24]. Nonetheless, a marginal benefit of AHSCT was suggested in cases with recent clinical or MRI inflammatory disease activity [22••, 25]. Compared to inactive cases, a sustained EDSS improvement for 3 years after transplant was reported in MRI-active (recent gadolinium-enhancing lesions) ‘newly diagnosed SPMS’ patients, defined as patients referred as RRMS but who, upon examination, indicated a gradual change in baseline neurologic disability starting within 2 years and independent of relapse activity [22••].

Similar outcomes were observed in monocentric studies including up to 30 RRMS [27, 28]. A substantial stabilisation in cognitive functions was reported in 13 RRMS patients treated with Cy-ATG protocol, with a transient improvement at month 12 in information processing speed and verbal learning, followed by stabilisation at month 24 compared to baseline [27]. A significant reduction in fatigue score and improvement in some domains (physical functioning, vitality and pain) of the short-form 36 health survey questionnaire were also described [29].

### Progressive MS: Retrospective Matched Studies 

Two retrospective studies compared SPMS patients treated with AHSCT with matched controls, including patients with a relatively short duration of the progressive phase (on average 2 [30] to 3 years [31]) and inflammatory features in most cases, although with moderate to severe disability (baseline EDSS: 6).

A multicentric study included 79 SPMS patients treated with AHSCT (81% BEAM-ATG protocol) and 1975 SPMS who had started treatment with DMTs (siponimod, cladribine and anti-CD20 monoclonal antibodies excluded) after the diagnosis of SPMS, selected from the Italian MS register using propensity score [30]. AHSCT was superior to DMTs on the outcomes: PFS (at year 5: 62% and 46%, respectively; hazard ratio (HR) 0.50), mean EDSS change over 10 years (− 0.013 and + 0.157 EDSS points/year, respectively), prevalence of disability improvement (at year 5: 19% and 4%, respectively) and reduction of ARR (over the entire follow-up: 0.020 and 0.45, respectively). The mean yearly EDSS accumulation was lower in AHSCT-treated patients compared to controls treated with either interferon beta-1b or mitoxantrone, DMTs approved in Italy for the treatment of SPMS (sensitivity analyses).

One monocentric study compared 31 AHSCT-treated SPMS patients (BEAM-ATG protocol) with 62 propensity-score-matched SPMS controls treated with pulses of Cy [31]. Complete suppression of relapse activity was observed in the AHSCT group only (RFS at year 5: 100% vs. 52%), even though Cy also reduced ARR significantly compared to pre-treatment (from 0.46 to 0.20). Rates of PFS were similar between groups (at year 5: 45% AHSCT vs. 48% Cy). Nonetheless, when evaluating the disability trajectory after the first episode of EDSS worsening, AHSCT-treated patients tended to have a lower risk of maintaining a progressive disease course (HR = 0.65) and achieving long-term severe disability compared to controls (6% vs. 18%), although the difference was not significant.

### Treatment-Naïve MS

One retrospective multicentric study reported the use of AHSCT as a first-line treatment in 20 aggressive MS patients with multiple clinical and radiological features suggestive of poor prognosis [32]. The treatment was performed early in the disease course, with a median interval between MS diagnosis and AHSCT of only 5 (range 1–20) months, and different transplant protocols were utilised (Table 1). Over a median follow-up of 30 (range 12–118) months, none of the patients experienced confirmed disability progression or relapses or new MRI activity after re-baselining at month 6. Furthermore, disability improved in 95% of the cases, with a median reduction of 2.25 (range 0–6.5) EDSS points, although this may be affected by the recent accrual of disability at baseline. As for safety, no grade 4 toxicities or transplant-related mortality (TRM) was reported; secondary autoimmune thyroiditis was observed in four patients (20%).

### Comparative Studies with High-Efficacy DMTs

Prospective comparisons between AHSCT and high-efficacy DMTs are currently lacking, as natalizumab only was included in the comparator arm of the MIST trial, and the number of treated cases was small [33].

Three retrospective cohort studies compared the use of AHSCT and alemtuzumab on RRMS [34•, 35] or RRMS and progressive MS patients [36•]. The sample size ranged from 40 [36•] to 144 patients [34•], and the AHSCT protocol used was either Cy-ATG [34•] or BEAM-ATG [35, 36•]. Due to possible selection biases intrinsic to the retrospective and uncontrolled design, baseline characteristics differed between groups, being that AHSCT-treated patients were generally younger (or with shorter disease duration), more disabled and inflammatory-active compared to alemtuzumab-treated patients; the post-treatment follow-up was also longer (up to more than two-fold) after AHSCT than alemtuzumab [35, 36•], except in [34•]. As these patient population differences were generally disadvantageous for AHSCT, the observed results may plausibly represent the lower boundary for the true effect of transplant.

NEDA-3 status was consistently and significantly higher after AHSCT than alemtuzumab in all the studies, ranging from 88% vs. 37% at year 3 [34•] to 62% vs. 40% at the end of the observation period (median 59 and 28 months in the AHSCT and alemtuzumab groups, respectively; *p* = 0.001) [36•]. When analysing individual components of NEDA, AHSCT was superior to alemtuzumab on relapses and new MRI activity, with a similar effect on disability progression in two studies [35, 36•], whereas it was superior also on PFSin the study with similar follow-up duration between groups [34•]. Disease activity in AHSCT-treated patients was determined exclusively by EDSS worsening in one study, where it was primarily observed in cases with progressive disease: notably, SPMS was included in both the AHSCT (4/19) and the alemtuzumab groups (5/21), but PPMS (3/19) were in the AHSCT group only [36•]. Interestingly, applying a rebaseline at 1 year after treatment initiation, AHSCT was still substantially and significantly superior to alemtuzumab on NEDA-3 survival, RFS and PFS, whereas MRI activity-free survival was similar between the groups [34]. In another study with MRI rebaseline at 1 year, AHSCT was still superior to alemtuzumabin this outcome [35].

Compared to alemtuzumab, AHSCT was associated with a higher probability of sustained improvement in EDSS [34•, 35, 36•] and cognitive outcomes [36•], with improved cognitive functions without any worsening in the short term (3-month assessment) after AHSCT, opposite to cases treated with alemtuzumab who deteriorated at follow-up in all the tested domains.

The comparative effectiveness of AHSCT vs. fingolimod, natalizumab and ocrelizumab in RRMS was explored in 167 AHSCT-treated patients who were propensity score matched to controls exposed to one of these DMTs, selected from the MSBase Registry [37]. Over 5 years, AHSCT was associated with a lower risk of relapses and a higher chance of disability improvement compared with fingolimod and natalizumab, with a similar effect on disability worsening. Over 3 years, the effect on relapses and disability outcomes was similar between AHSCT and ocrelizumab, but the follow-up for the latter was of mean 1.5 years only. Besides the shorter follow-up for the ocrelizumab group, limitations of the study included the use of different AHSCT protocols, possible residual heterogeneity in patient populations, lack of data on MRI activity and potential ascertainment bias due to different follow-up schedules between the AHSCT and DMT groups.

### Costs 

An exploratory study estimated the relative effectiveness of AHSCT versus natalizumab in RRMS using a matching-adjusted indirect comparison informed by data from (i) a cohort of patients treated with AHSCT within European transplant centres and (ii) outcome data relating to the intervention arm of the AFFIRM trial of natalizumab [38]. The HR for sustained EDSS progression for AHSCT versus natalizumab was estimated to be 0.11 (95% confidence interval 0.02, 0.76), suggesting that AHSCT may be highly clinically effective for the treatment of RRMS and that it may represent a cost-effective use of health care resources, given its once-only nature compared to the substantial lifetime costs of continuous treatment using DMTs.

Two studies analysed the costs of AHSCT in two different healthcare systems [39, 40]. In the US study, mean total costs of AHSCT were $85,184, whereas DMT costs from the literature ranged from $80,000 to $100,000 per year per patient [39]. As studies of AHSCT reported greater improvement in efficacy outcomes compared to those of DMTs, the authors concluded that AHSCT may be a ‘win–win’ in terms of both cost and clinical efficacy, possibly capable of generating cost savings and additional health gains for well-selected RRMS patients compared with standard DMTs. In the Polish study, costs of AHSCT were estimated at around €12,000 [40]. When analysing costs covered by the National Health Fund for the 105 patients treated with AHSCT over the study period, mean treatment-related costs per patient-year before and after the transplantation were €4315 and €1189, respectively. Even if the latter rose to a mean value of €6295 when including the costs of AHSCT, the transplant induced a reduction of all treatment costs by 82% and payed off its costs in 3.9 years.

## Safety 

### Treatment Sequencing

Treatment sequencing with AHSCT may be challenging after discontinuation of DMTs with long-standing effects on the immune system, as it requires the identification of adequate wash-out periods that allow a safe transition to AHSCT [4], minimising in the meantime the risks for disease reactivation, especially after the withdrawal of lymphocyte-sequestering DMTs [41]. In this respect, no safety issues were recently reported in 26 patients who received alemtuzumab, rituximab or cladribine in the last 6 months before Cy-based AHSCT compared to the 78 cases who had received standard DMTs or no treatment, with similar times to engraftment and risk of neutropenic fever and secondary autoimmunity [23]. These observations suggest that performing AHSCT within 6 months from such DMTs is safe, although the number of patients receiving each DMT was small, and the wash-out duration was not reported for every treatment. A careful and comprehensive evaluation of the individual risk profile is therefore required when adopting short wash-out periods.

### Secondary Autoimmunity

Risks for secondary autoimmunity after treatment with different conditioning regimens were recently reviewed in [42], where it was high with busulfan-based (18%) and low-moderate with non-myeloablative regimens (7.7%), except for those containing alemtuzumab, being higher (14%). Pooled rates of secondary autoimmunity were below 1% after BEAM-AHSCT [42], but this was plausibly due to under-reporting: more recently, a similar risk was described between BEAM-ATG and Cy-ATG, with an almost six-fold increase in autoimmune thyroiditis in 139 patients who received AHSCT with either of these two regimens (incidence rate (IR) per 1000 person-years 34) compared to a matched population of MS patients who received non-induction therapies (IR 5.3) [43•].

In recent cohort studies, the incidence of secondary autoimmunity ranged from 6% [24] to 17% [28]. The use of alemtuzumab in the conditioning regimen was associated with a higher risk for idiopathic thrombocytopenic purpura compared to the use of ATG (alone or in combination with intravenous immunoglobulins or rituximab) in one study (11.5% vs. 2–3%, respectively), whereas the risk for thyroiditis was similar (roughly 10%) [22••]. In retrospective comparative studies, a two- to three-fold higher risk for secondary autoimmunity was observed in alemtuzumab-treated compared to AHSCT-treated patients [34•, 35,43•].

### Impairment of Gonadal Function and Fertility 

Recent studies expanded the knowledge on the impact of AHSCT on gonadal function and fertility, which was previously mainly derived from studies on haematological patients [44, 45]. In females undergoing AHSCT for MS, persistent amenorrhea ranged from 30% with mixed conditioning regimens (BEAM-ATG or Cy-ATG) [46] to 43% with Cy-ATG protocol [28]. Older age at AHSCT and prior use of Cy were predictors of persistent amenorrhea in one study, where no differences were detected between BEAM-ATG and Cy-ATG regimens, although the sample size was small [46].

Anti-mullerian hormone (AMH) concentration, a marker of ovarian reserve, was largely decreased compared to baseline in females transplanted with Cy-ATG protocol [47]. Nonetheless, mense resumption [46] and spontaneous pregnancies [47] were observed in women with post-treatment AMH levels lower than those expected for age.

Successful spontaneous pregnancies/conceptions without newborn complications were reported in a few females and males after either BEAM-ATG [46, 48] or Cy-ATG protocols [28, 46, 47], even in females showing amenorrhea or oligomenorrhea [48]. Successful pregnancies were reported in three of four women who tried to conceive after transplant in one study [46], but the actual pregnancy rate after AHSCT in MS cannot be estimated due to the lack of systematic assessment of desire for pregnancy.

Hormonal replacement therapy is usually recommended after AHSCT in women with premature ovarian failure; to our knowledge, no recommendations are currently available on the use of contraception after AHSCT in the autoimmune setting.

### Transplant-Related Mortality

TRM ranged from 0.19% [22••] to 2.5% [24] in cohort studies, and it was close to, or equal to, 0% in most recent studies [23, 27, 31, 32, 34•, although its reliable estimation should be performed in wide patient cohorts or registry studies only.

In the population-based cohort study from Sweden, the IR of mortality after AHSCT was 1.7 (95% CI 0.0–9.6) per 1000 person-years (1 suicide) compared with 8.6 (2 suicides, 1 heart attack, 1 CMV reactivation) and 0.7 in the alemtuzumab group and reference population, respectively [43•].

## Limitations of Current Studies and Upcoming RCTs for AHSCT

Comparisons between studies are limited by heterogeneity in study design, AHSCT protocol, inclusion criteria and definition of treatment failure. Even if comparative studies on the effectiveness of different AHSCT protocols are lacking, it is plausible that the use of high-intensity regimens may be more effective than lower-intensity regimens in suppressing new inflammatory activity, and this question should be addressed in future studies. Besides the protocol used, patient selection is a key determinant of both efficacy and safety outcomes after AHSCT: as an example, the inclusion of different proportions of early active RRMS vs. late-stage progressive MS patients affects PFS, which is highest in early RRMS. Indirect comparisons may be further limited by heterogeneity in the definition of treatment failure, such as the use of different cut-offs for disability progression.

Heterogeneity may also be observed across upcoming RCTs, mainly related to the AHSCT protocol used, eligibility criteria and DMTs administered in the comparator arm; while harmonisation could reduce the variables, some heterogeneity (e.g. different conditioning protocol intensity) could provide valuable complementary information.

## Conclusions 

Recently published studies expanded the knowledge on several aspects concerning AHSCT in MS, providing confirmatory evidence on known mechanisms of action, as well as new insights towards the identification of novel biomarkers of treatment response. Large cohort studies confirmed optimal outcomes in RRMS, and retrospective comparative studies showed possible superior effectiveness over alemtuzumab. A marginal benefit was suggested in progressive disease, especially in early SPMS with inflammatory features, but the potential role of AHSCT in this form is yet to be defined. New data on the impact of AHSCT on fertility and secondary autoimmunity were also provided.

In conclusion, recent evidence reinforces the role of AHSCT as a clinical option in aggressive RRMS, on grounds of high effectiveness and acceptable safety profiles. Further research is needed to better define its role in treatment-naïve and progressive MS, preferably in the context of RCTs.
